# Supplementary material for: Female Gender Is Associated with an Increased Left Ventricular Ejection Fraction Recovery in Patients with Heart Failure with Reduced Ejection Fraction
Source: Med Sci (Basel). 2022 Apr 2;10(2):21. doi: 10.3390/medsci10020021 (PMC9036235; doi:10.3390/medsci10020021)
Supplement: Supplementary file 1 [file medsci-10-00021-s001.zip › medsci-1587067-supplementary.pdf]

Search term

Medline

("heart failure"[MeSH Terms] OR ("heart"[All Fields] AND "failure"[All Fields]) OR "heart failure"[All Fields]) AND (("recover"[All Fields] OR "recovered"[All Fields] OR "recovering"[All Fields] OR "recovers"[All Fields]) AND ("eject"[All Fields] OR "ejected"[All Fields] OR "ejecting"[All Fields] OR "ejection"[All Fields] OR "ejectional"[All Fields] OR "ejections"[All Fields] OR "ejects"[All Fields]) AND ("dose fractionation, radiation"[MeSH Terms] OR ("dose"[All Fields] AND "fractionation"[All Fields] AND "radiation"[All Fields]) OR "radiation dose fractionation"[All Fields] OR "fractionation"[All Fields] OR "chemical fractionation"[MeSH Terms] OR ("chemical"[All Fields] AND "fractionation"[All Fields]) OR "chemical fractionation"[All Fields] OR "fraction"[All Fields] OR "fraction s"[All Fields] OR "fractionate"[All Fields] OR "fractionated"[All Fields] OR "fractionates"[All Fields] OR "fractionating"[All Fields] OR "fractionationed"[All Fields] OR "fractionations"[All Fields] OR "fractionator"[All Fields] OR "fractionators"[All Fields] OR "fractioned"[All Fields] OR "fractioning"[All Fields] OR "fractionized"[All Fields] OR "fractions"[All Fields]))

EMBASE

('heart failure'/exp OR 'heart failure' OR (('heart'/exp OR heart) AND ('failure'/exp OR failure))) AND ('recovered ejection fraction' OR (recovered AND ejection AND fraction)) AND ([article]/lim OR [article in press]/lim)

Table S1: Newcastle-Ottawa Scale of the included studies.

First author, year	Selection				Comparability	Outcome			Total score
	Representativeness	Selection of the non-exposed cohort	Ascertainment	Endpoint not present at start	Comparability (Confounding)	Assessment of outcome	Follow-up duration	Adequacy follow-up	
Abe, 2020	*	*	*	*	**	*	*	*	9
Basuray, 2014	*	*	*	*	**	*	*	*	9
Bermejo, 2017	*	*	*	*	**	*	*	*	9
Chang, 2018	*	*	*	*	**	*		*	8
Chang, 2020	*	*	*	*	**	*	*	*	9
Florea, 2016	*	*	*	*	**	*	*	*	9
Howlett, 2020	*	*	*	*	**	*	*	*	9
Kalogeropoulos, 2016	*	*	*	*	**	*	*	*	9
Lupon, 2017	*	*	*	*	**	*	*	*	9
Pereira, 2019	*	*	*	*	**	*	*	*	9
Punnoose, 2011	*	*	*	*	**	*	*	*	9
Shah, 2020	*	*	*	*	**	*	*	*	9
Swat, 2018	*	*	*	*	**	*	*	*	9
Trullas, 2016	*	*	*	*	**	*	*	*	9
Torii, 2021	*	*	*	*	**	*	*	*	9
Ye, 2021	*	*	*	*	**	*			7
Zeller, 2021	*	*	*	*	**	*	*	*	9
Zhang, 2021	*	*	*	*	**	*	*	*	9

The Newcastle-Ottawa scale uses a star system (0 to 9) to evaluate included studies on 3 domains: selection, comparability, and outcomes. Star (*) = item presents. Maximum 1 star (*) for selection and outcome components and 2 stars (**) for comparability components. Higher scores represent higher study quality.
